# Unexpected Electron Transport Suppression in a Heterostructured Graphene–MoS_2_ Multiple Field-Effect Transistor Architecture

**DOI:** 10.1021/acsnano.1c09131

**Published:** 2021-12-23

**Authors:** Gaia Ciampalini, Filippo Fabbri, Guido Menichetti, Luca Buoni, Simona Pace, Vaidotas Mišeikis, Alessandro Pitanti, Dario Pisignano, Camilla Coletti, Alessandro Tredicucci, Stefano Roddaro

**Affiliations:** † Dipartimento di Fisica “E. Fermi”, Università di Pisa, Largo B. Pontecorvo 3, I-56127 Pisa, Italy; ‡ Graphene Labs, Istituto Italiano di Tecnologia, Via Morego 30, I-16 163 Genova, Italy; § NEST, CNR—Istituto Nanoscienze and Scuola Normale Superiore, Piazza San Silvestro 12, I-56 127 Pisa, Italy; ∥ Center for Nanotechnology Innovation @NEST, Istituto Italiano di Tecnologia, Piazza San Silvestro 12, I-56 127 Pisa, Italy

**Keywords:** graphene, MoS_2_, heterostructure, field-effect, single-crystal

## Abstract

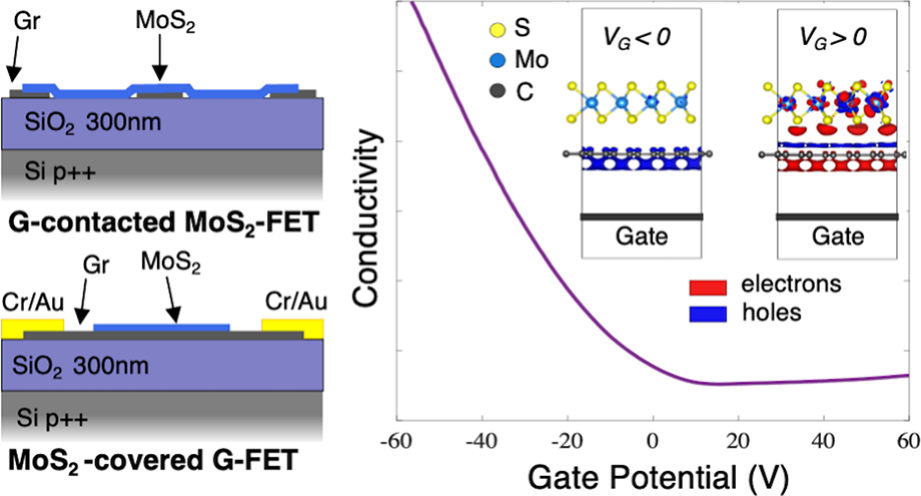

We demonstrate a graphene–MoS_2_ architecture integrating multiple field-effect transistors (FETs), and we independently probe and correlate the conducting properties of van der Waals coupled graphene–MoS_2_ contacts with those of the MoS_2_ channels. Devices are fabricated starting from high-quality single-crystal monolayers grown by chemical vapor deposition. The heterojunction was investigated by scanning Raman and photoluminescence spectroscopies. Moreover, transconductance curves of MoS_2_ are compared with the current–voltage characteristics of graphene contact stripes, revealing a significant suppression of transport on the *n*-side of the transconductance curve. On the basis of *ab initio* modeling, the effect is understood in terms of trapping by sulfur vacancies, which counterintuitively depends on the field effect, even though the graphene contact layer is positioned between the backgate and the MoS_2_ channel.

Formed when two or more atomically thin crystals are bonded by van der Waals (vdW) interaction,^1^ vdW heterostructures are intriguing architectures, enabled by the discovery of two-dimensional (2D) materials, such as graphene, hexagonal boron nitride, and transition metal dichalcogenides (TMDs). Within this family, peculiar junctions can be obtained when graphene is used as a contact material for a TMD monolayer. While the interface between a TMD and a conventional, bulk metallic electrode tends to display Schottky behavior due to intrinsic and extrinsic Fermi pinning phenomena,^2−4^ vdW graphene–TMD junctions yield well-behaved linear transport characteristics.^5^ This contacting approach has been successful in improving TMD-based devices,^6^ and together with the side-contacting approach,^7−9^ it is commonly used for the realization of most devices based on 2D materials. Graphene–TMDs heterostructures were employed for many other applications, e.g., in flexible photodetectors.^10^ Nevertheless, the exact physics behind graphene–TMD vdW junctions is still debated^11−13^ and difficult to probe in a direct way. In particular, devices typically include only two contacts, which makes the effects on the transport characteristics due to the interface not easy to distinguish from those due to the resistivity of the 2D materials, since typically only the global conductance of the device can be measured. Charge transfer phenomena,^14^ strain,^15^ and charge trapping in defects^16−18^ might also play an important role. Furthermore, in the case of field-effect devices, the low density of states in the vicinity of the Dirac point leads to weak screening properties despite the metallic nature of graphene.^19^ This implies that a nontrivial response to field effect can be observed and exploited in device concepts.^20,21^ Gating on graphene–MoS_2_ heterostructures has been widely investigated from a numerical^22^ and experimental^5,23−25^ point of view and in different stacking configurations. Indeed, the reciprocal electrostatic screening of the junction materials can affect the contact resistance, and it was shown theoretically that MoS_2_ can screen the field effect on graphene, or not, depending on the order of the specific stacking sequence.^22^ Nevertheless, to the best of our knowledge, direct experimental evidence of how the formation of vdW interfaces leads to changes in the transport properties of the individual materials involved in field-effect transistor (FET) devices is still missing.

## Results and Discussion

**Figure 1:**
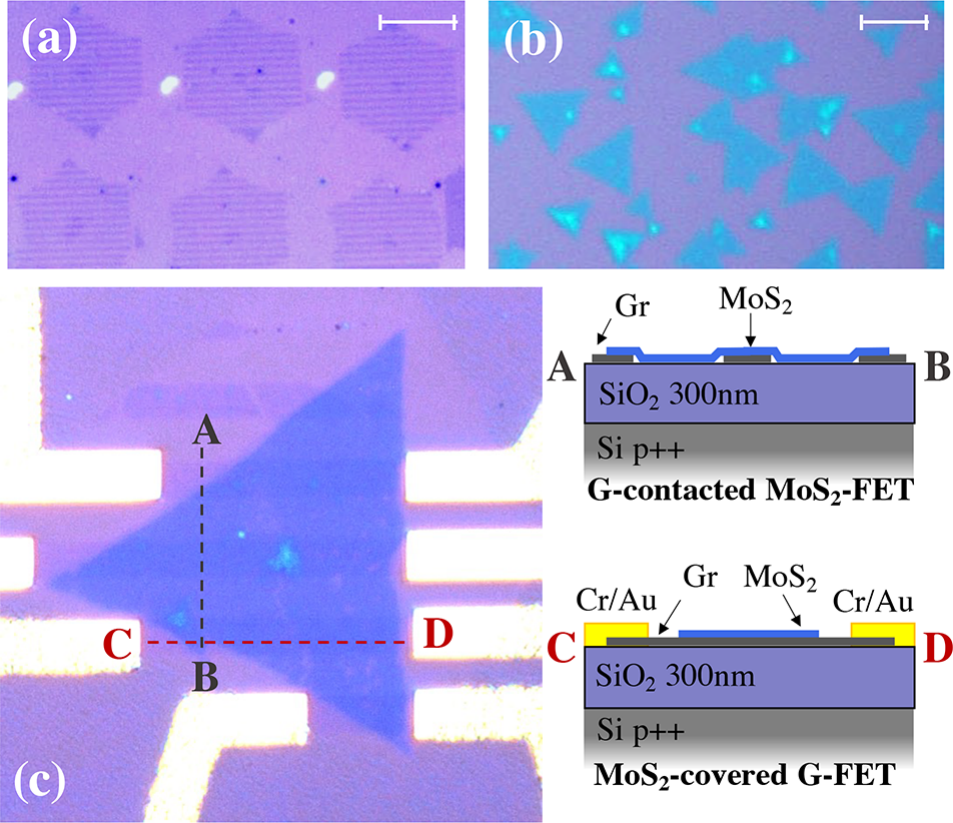
Multi-FET device architecture. (a) Monocrystalline contact stripes obtained by patterning a periodic array of graphene CVD flakes (scale bar is 100 μm). (b) Monocrystalline CVD MoS_2_ flakes before transfer onto the SiO_2_/Si substrate (scale bar is 50 μm). (c) Optical picture of one of the studied devices implementing a multiple FET structure, as visible in the cross section sketches: graphene multicontact MoS_2_ FET (AB section) and MoS_2_-covered graphene FET (CD section).

The progress of large-scale chemical vapor deposition (CVD) techniques gives us the opportunity to investigate vdW interfaces from a different angle. High-quality and large-scale monocrystalline flakes of graphene^26,27^ and TMDs^28,29^ can be reproducibly grown. When this technique is associated with a patterning of the seed points, predictable flake arrays of chosen sizes can be achieved,^30^ enabling the fabrication of multiple parallel devices combining different 2D materials. Here, we take advantage of this opportunity to demonstrate a graphene–dichalcogenide architecture where a monocrystalline MoS_2_ channel is contacted by a large number of monocrystalline graphene stripes, each of them crossing the whole MoS_2_ channel as schematized in Figure 1. Each stripe can thus act as ohmic contact for a MoS_2_ backgated FET (see cross section AB in Figure 1) and be simultaneously contacted at its terminations to implement an additional MoS_2_-covered graphene FET (cross section CD in Figure 1). This structure can so act as a MoS_2_ FET and as a set of graphene FETs at the same time, which will be referred to as a *multi-FET* in the following.

Such an arrangement allows us to study the conducting properties for the different components of graphene–MoS_2_ systems, highlighting the suppression of the electron-side transconductance in MoS_2_-covered graphene, in coincidence with the conducting threshold of free MoS_2_ in the main device channel. This behavior is apparently at odds with recent predictions for defect-free MoS_2_;^22^ nonetheless, it can be understood in terms of a gate-driven trapping by sulfur vacancies which further highlights the nontrivial weak screening properties of graphene.

**Figure 2:**
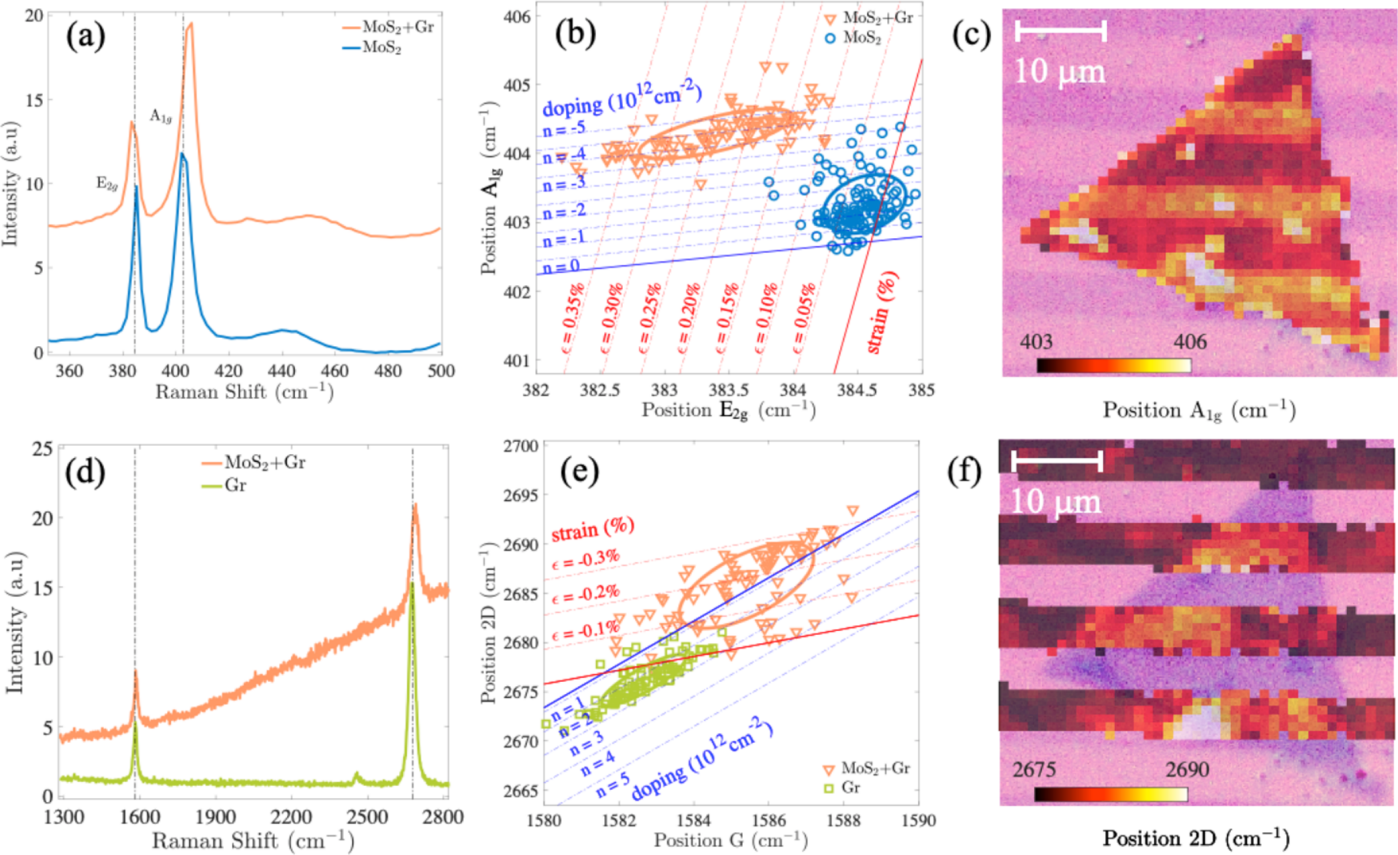
Raman measurements of the MoS_2_–graphene structures. (a) MoS_2_ Raman spectra after transfer on top of the graphene stripes: both spectra from MoS_2_ on top of graphene (orange) and graphene-free MoS_2_ (blue) are reported. (b) Correlation plot of the position of A_1g_ as a function of the position of E_2g_. Zero-strain and zero-doping lines are taken from ref (31) (514.5 nm laser excitation). (c) Map of the position of A_1g_. (d) Raman spectra of graphene after the MoS_2_ transfer: both spectra in the presence (orange) and absence (green) of the MoS_2_ overlayer are reported. (e) Correlation plot of the position of 2D peak as a function of the position of G peak. Zero-strain and zero-doping lines are taken from ref (32) (514.5 nm laser excitation). (f) Map of the position of the 2D peak. Correlation plots in panels b and e were obtained from Raman spectra collected as far as possible from the flakes boundaries to avoid spillover effects from neighboring regions and do not derive from the data sets used in panels c and f.

**Figure 3:**
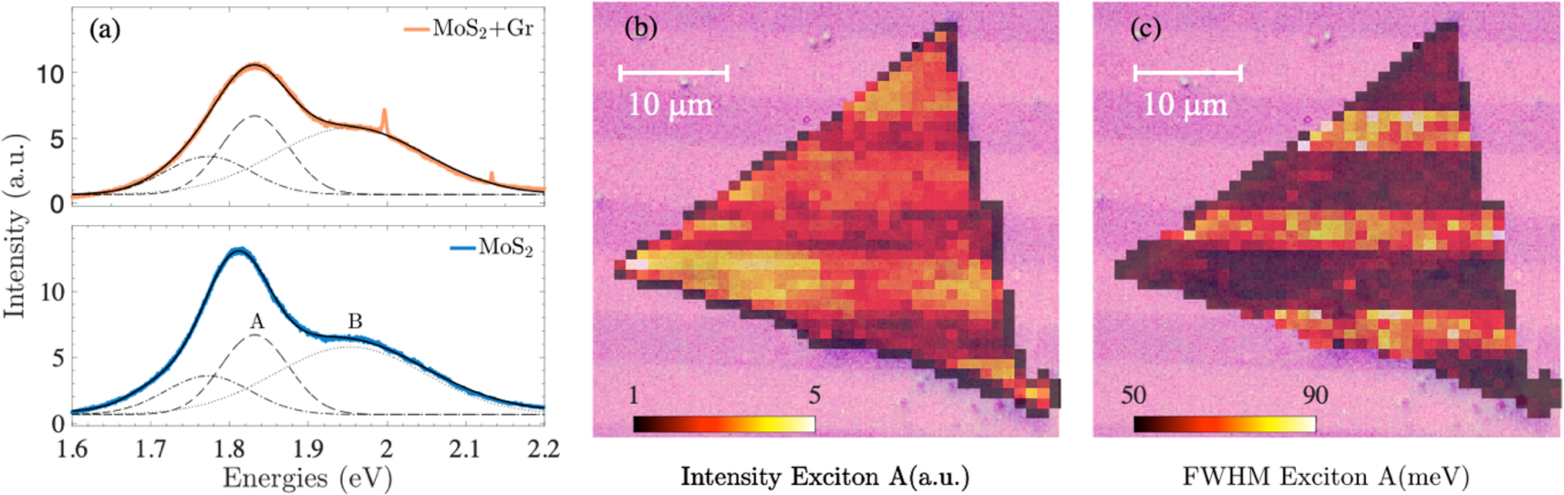
Photoluminescence measurements of the MoS_2_–graphene structures. (a) PL spectra of MoS_2_ both in a region where it overlaps graphene (orange) and in a graphene-free region (blue). Gaussian fits of A and B excitons are shown in dashed and dotted lines, respectively. (b) Map of the position-dependent quenching of the A exciton signal. (c) Map of the position-dependent A exciton broadening. All maps are shown in overlay to an optical image of the analyzed flake; scale bars in the panels correspond to 10 μm.

Both the patterning procedures and the formation of vdW interfaces can significantly perturb the properties of the 2D materials. For this reason, photoluminescence (PL) and Raman spectroscopy were employed to characterize the 2D crystals at relevant device processing steps. In Figure 2a–c, we report Raman spectra of a typical transferred MoS_2_ flake and analyze the influence of the graphene contact stripes. We analyze a MoS_2_ flake with a small bilayer island both in regions with and without graphene. The Raman spectra of bilayer MoS_2_–graphene heterostructure and bare MoS_2_ bilayer are reported in the Supporting Information. The characteristic A_1g_ and E_2g_ modes visible in Figure 2a exhibit a strong dependence on thickness,^33^ and their separation of Δω ≃ 19 cm^–1^ is in good agreement with the expected monolayer nature of the MoS_2_ flake. The shift of the A_1*g*_ mode in Figure 2b,c can be interpreted as caused by the interlayer interaction between MoS_2_ and graphene, as reported in ref (34). Nevertheless, a recent work^35^ proposes an alternative interpretation in terms of doping and strain, extending the scope of a method which is typically used for bare graphene^32^ and bare MoS_2_.^31^ The following discussion is based on this last interpretation. Starting from the strain and doping reference lines reported in ref (31), we consider the correlation plot in Figure 2b, where the position of the A_1g_ peak is plotted against the one of E_2g_.^31^ The mean Raman shifts of the E_2g_ and A_1g_ peaks in graphene-free regions are 384.5 ± 0.3 and 403.3 ± 0.4 cm^–1^, respectively. These values are quite close to the neutrality point, located at the intersection between the zero strain and zero doping lines (E_2g_ = 384.6 ± 0.2 cm^–1^ and A_1g_ = 402.7 ± 0.2 cm^–1^) .^31^ Interestingly, Raman shifts from regions where MoS_2_ overlaps graphene (E_2g_ = 383.4 ± 0.5 cm^–1^ and A_1g_ = 404.3 ± 0.3 cm^–1^) indicate a variation in both tensile strain distribution of ≈0.10–0.35% and a sizable electron reduction of (3.0 ± 1.8) × 10^12^ cm^–2^. Thanks to the sensitivity of the A_1g_ peak on doping,^36^ the spatial doping modulation of MoS_2_ due to graphene can be directly appreciated in the map of the A_1g_ position in Figure 2c. The map is shown in overlay to an optical picture of the flake, to highlight the good correlation between the map patterns and the position of the graphene stripes. Consistent evidence is obtained from the graphene Raman data shown in Figure 2d–f. In Figure 2d, the graphene spectra in the presence/absence of MoS_2_ are compared. Both curves show a single sharp Lorentzian-shaped 2D peak, which is a typical signature of monolayer graphene,^37^ and no D peak, which indicates a negligible density of defects.^38^ The absence of defects was confirmed for all the fabrication steps (see the Supporting Information). We also note that when graphene is covered in MoS_2_ (orange curve), a strong baseline appears below the Raman peaks due to the MoS_2_ PL signal. As in the case of MoS_2_, the strain and doping profiles can be derived from the Raman data,^32^ based on the correlation plot of the 2D and G modes reported in Figure 2e, where the strain and doping reference lines are taken from ref (32) (see Supporting Information for additional correlation plots). The positions of the G and 2D peaks in regions free from MoS_2_ are 1582.7 ± 0.9 and 2676.2 ± 2.2 cm^–1^, respectively, corresponding to a p-type doping. In contrast, Raman data collected in MoS_2_-covered regions (1585.4 ± 1.5 and 2685.8 ± 3.7 cm^–1^ for the G and 2D peaks, respectively) fall on the strain line, thus indicating a neutralization of graphene. The variation of mean peaks position corresponds to an electron increase of (2.3 ± 1.5) × 10^12^ cm^–2^ and to a variation of strain nature from tensile to compressive. The spatial modulation of the doping can be seen from the 2D peak position map in Figure 2f, showing a good correlation with the position of the MoS_2_ flake. A modified strain is also observed, turning from slightly tensile to compressive, ≈ 0.10–0.30%. We note that the doping and strain trends observed where MoS_2_ and graphene overlap are opposite and are thus consistent. We further highlight that while the discussed analysis of the Raman data indicates an electron transfer from MoS_2_ to graphene^35^ the absolute equilibrium carrier densities are not obvious to quantify. This is due to the presence of photoexcited carriers during the Raman measurements, and to the unknown exact calibration of the zero-strain and zero-doping points (standard values from refs (31) and (32) were used). The formation of the heterojunction can be further investigated based on the PL spectra of MoS_2_, which are reported in Figure 3a. Three main peaks are highlighted by Gaussian deconvolution. These peaks are attributed to the A exciton (1.81 eV), the B exciton (1.94 eV), and the trion (1.71 eV).^39,40^ We observe that the presence of graphene modifies the MoS_2_ response and that the signal of the A exciton is quenched when MoS_2_ is coupled to graphene (orange curve) with respect to stand-alone MoS_2_ (blue curve): indeed, a lowering of the A intensity by ∼30% and a line shape broadening from ∼66 meV to ∼96 meV is retrieved. The spatial modulation of the effect can be directly appreciated from the maps of the intensity and width of the A exciton in Figure 3b,c, respectively. Additional spectroscopic data are reported in the Supporting Information.

**Figure 4:**
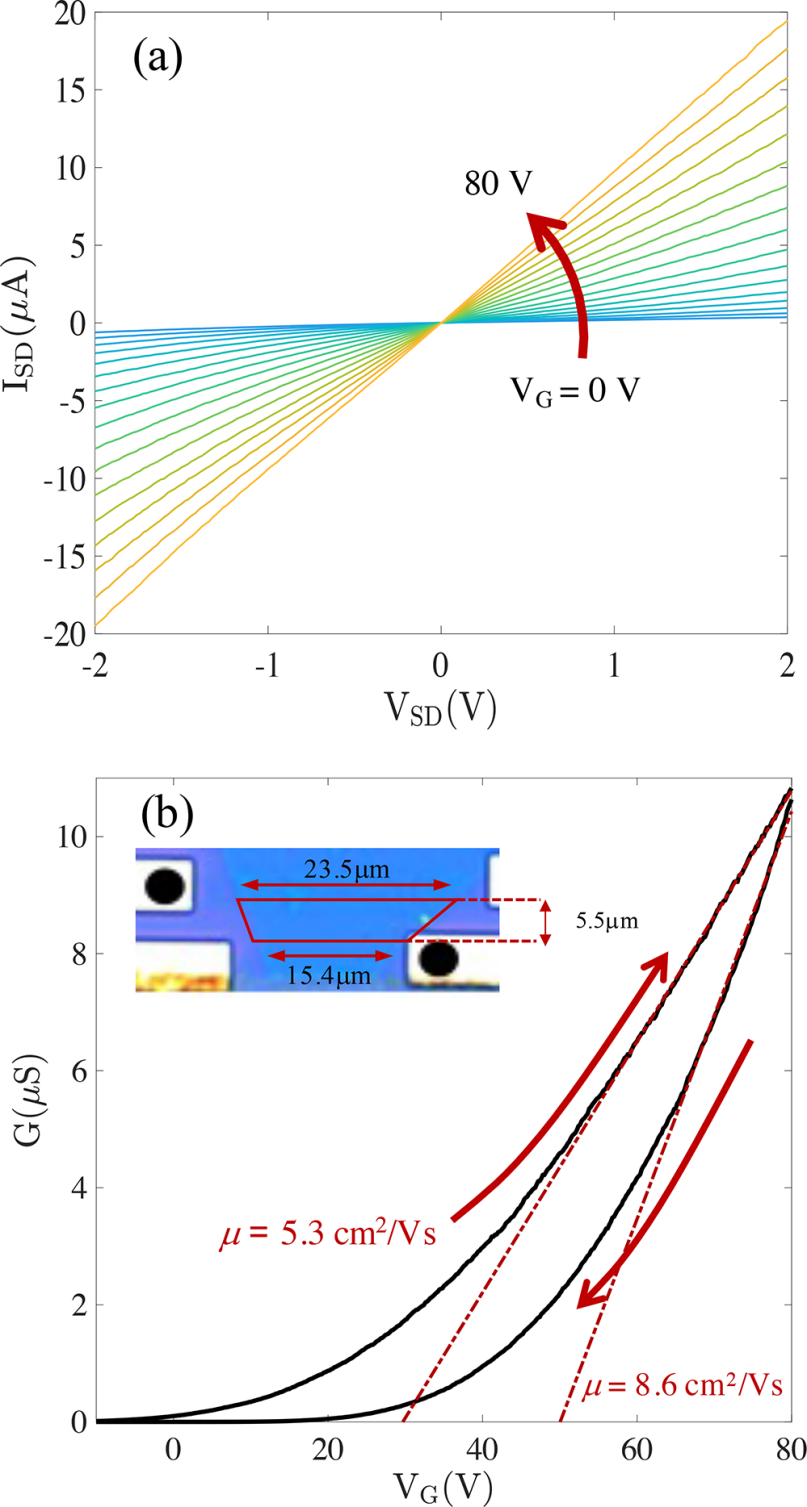
Transport characteristics of MoS_2_. (a) Room-temperature *I*–*V* characteristics of the MoS_2_ channel as a function of the gate voltage *V*_G_ in the 0–80 V range. (b) Transfer characteristics show a strong hysteresis, with red arrows indicating the sweep direction. Red dashed lines are the linear fits used to estimate the field-effect mobility for each of the two curves. Inset: an optical image of the measured device, with a sketch of the channel geometry and contacts highlighted by black dots.

The graphene stripes form ohmic contacts to the MoS_2_ channel and lead, at room temperature and in vacuum conditions (*P* < 10^–5^ mbar), to highly linear two-wire *I*–*V* curves over the ±2 V range. In Figure 4a, we report the *I*_SD_ versus *V*_SD_ characteristics of a representative MoS_2_ FET, measured as a function of the gate voltage (*V*_G_) in the 0–80 V range. The transfer characteristic in Figure 4b indicates a positive threshold voltage, with a sizable clockwise hysteresis, as frequently reported in the literature for FETs based on 2D materials and nanowires,^41−45^ as well as in Kelvin probe microscopy experiments.^46^ The effect is generally ascribed to the slow dynamics of trap states leading to a time-dependent screening of the field effect of the gate. Trap states may have several origins, including defects at the SiO_2_ substrate interface,^45^ adsorbates,^44^ or MoS_2_ point defects.^43^ In our devices, possible sources of traps include interfaces between MoS_2_, graphene, and SiO_2_ (see the AFM data in the Supporting Information), as well as S vacancies in MoS_2_, which are known to occur in quite large densities (typically few 10^13^ cm^–2^) in CVD flakes.^47,48^ The field-effect mobility of the MoS_2_ carrier can be estimated from the transfer characteristic according to
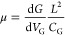
1where *C*_G_ is the capacitance and the MoS_2_ trapezoid channel sketched in the inset of Figure 4b is approximated as a rectangle with a length *L* = 5.5 ± 0.3 μm and width *W* = 19.5 ± 0.5 μm. Considering both curves in the hysteresis loop, we extract two mobility values, and similar analysis on different FETs yielded field-effect mobilities in the range of 5.3–6.6 cm^2^/(V s). Given that gate hysteresis generally indicates that part of the gate-induced carriers end in charge traps, field-effect measurements are known to overestimate the carrier density induced in the channel and to underestimate mobility.^42,49^ Both the mobility values above should thus be considered as a lower bound to the true room-temperature electron mobility in the specific MoS_2_ flake. The method also neglects the effect of contact resistances, which may lead to a mobility underestimation but are not expected to have a significant effect in the explored transport regime, based on preliminary four-wire measurement data.

**Figure 5:**
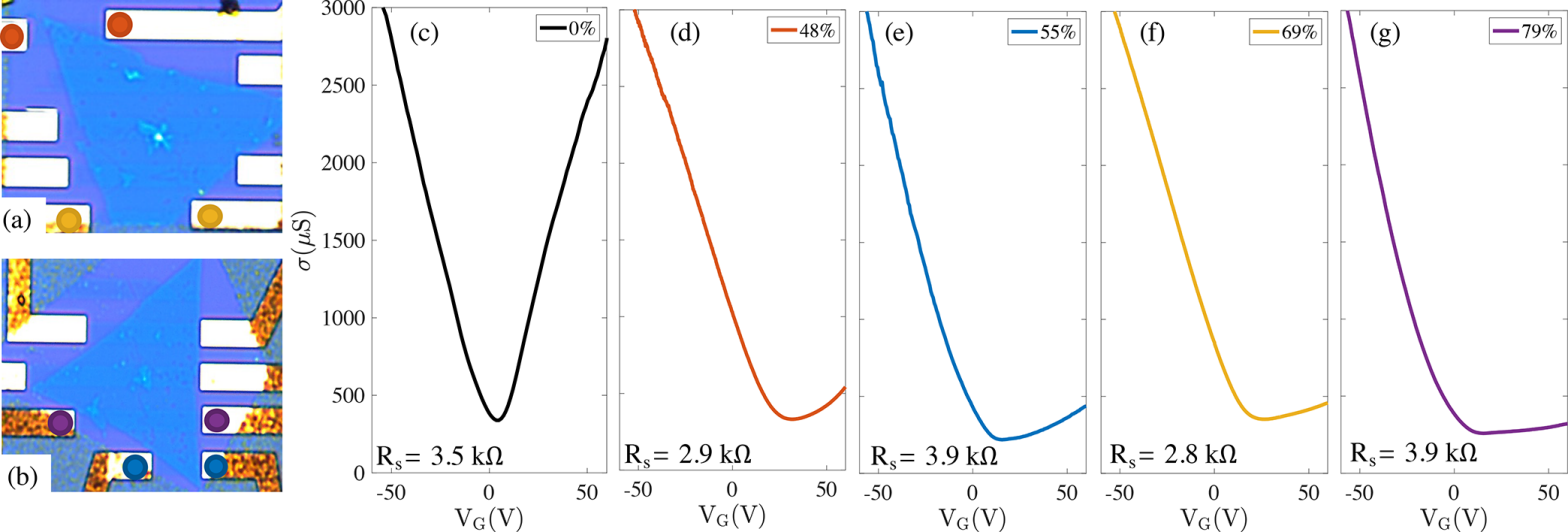
Effect of the MoS_2_ overlayer on electron transport in the graphene contact stripes. (a, b) Optical images of the two devices used to estimate the effect of different MoS_2_ coverage levels on conduction in the graphene stripes. Used contacts are highlighted by colored dots. (c–g) Transfer characteristics of the graphene stripes for different MoS_2_ coverages ranging from 0 to 79%. The curve colors match the ones used to highlight the contacts in panels (a) and (b): red, 48%; blue, 55%; yellow, 69%; and purple, 79%; the black curve corresponds to a reference MoS_2_-free graphene stripe (device image not shown).

Our multi-FET devices were specifically designed for comparing the MoS_2_ transport characteristics with the electron configuration in the graphene stripes, which play here the dual role of the contact in the MoS_2_ FET and of the channel in MoS_2_-covered graphene FETs. The *I*–*V* curves of all our graphene stripes are found to be highly linear (a representative measurement is reported in the Supporting Information), and in Figure 5 we report the transfer characteristic of various graphene FETs as a function of the *V*_G_, from which we obtain a mobility. These measurements are carried out at a fixed *V*_SD_ (0.2 V), as a function of *V*_G_. In the plot sequence of Figure 5c–g, we compare the conductivity of stripes characterized by a different MoS_2_ coverage: Conductivity is calculated from the total resistance using the geometrical form factor of the stripe, and contact resistances are estimated by comparing the p-side of the gate sweeps. MoS_2_ coverage is quantified from the ratio between the area of the MoS_2_–graphene and the graphene regions; see the optical pictures in Figure 5a,b. Coverage goes from 0% (MoS_2_-free graphene in Figure 5c) to 79% (Figure 5g). A clear trend is observed in the transfer characteristics: curves go from a conventional ambipolar behavior in Figure 5c to a limit of strongly quenched n-type conduction for the largest coverage in Figure 5g. The observation of a quenching of the field effect in graphene–TMD heterostructures has been reported in the literature, for instance in both WS_2_ and MoS_2_–graphene heterostructures and in graphene functionalized with different materials, such as TiO_2_ or organic molecules.^10,17,18,50−52^ The important role of MoS_2_ on electron transport suppression is clear as no deep suppression is observed in bare graphene stripes on SiO_2_ devices.^53^ However, the observed behavior is somewhat puzzling since, ideally, MoS_2_ should not affect carrier density in graphene when positioned on top of back-gated graphene due to the reciprocal screening in the vdW heterostructure.^22^

We ascribe the origin of this apparent discrepancy as due to vacancies in TMDs.^47,48^ This is a reasonable assumption, since we mentioned above that another possible source of trap states could be the SiO_2_ substrate. Nevertheless, the effect of the SiO_2_ substrate is secondary. In fact, the not-covered graphene stripe reported in Figure 5c has a standard symmetric behavior despite the presence of the SiO_2_ substrate. Moreover, the major contribution to the electron transport suppression from the sulfur vacancies can be deduced from the behavior reported in Figure 5c–g where a clear trend can be observed: The suppression increases as the MoS_2_ coverage increases. Furthermore, it is worth noting that the electrical transport of exfoliated graphene covered by exfoliated MoS_2_ usually does not present this suppression of the electron transport.^54^ In order to corroborate our hypothesis, and highlight the effect of sulfur vacancies, we perform density functional theory (DFT) calculations.

**Figure 6:**
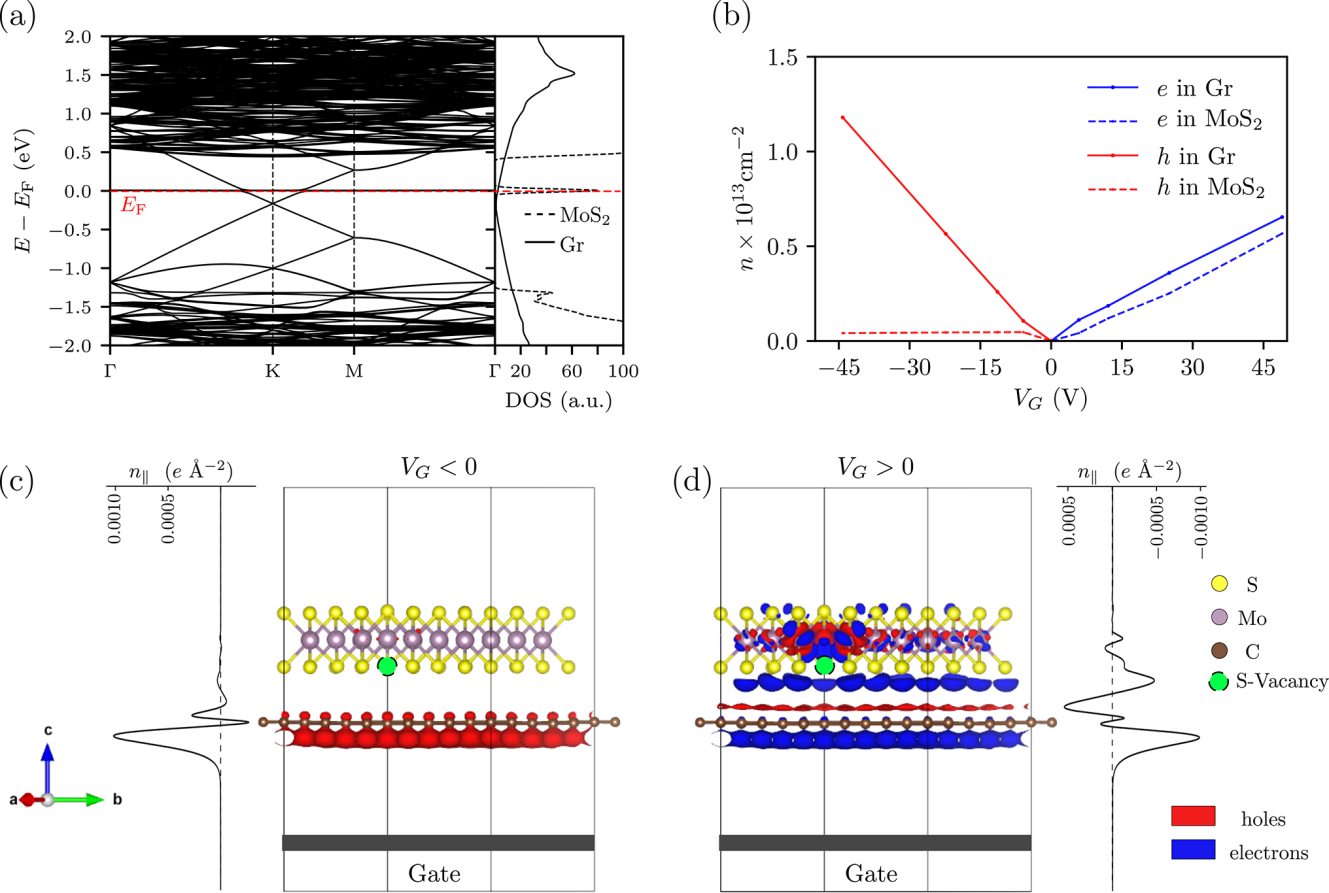
Field-effect response in the presence of S-vacancies. The impact of sulfur vacancies was simulated by removing one S atom from a MoS_2_ supercell (density of S-vacancies of ρ_v_ ≈ 1.8 × 10^13^ cm^–2^). (a) Supercell band structure and projected density of states (DOS) of the graphene–MoS_2_ interface (*V*_G_ ≈ 23 V corresponding to a charge induced by field effect *n* ≈ −6 × 10^12^cm^–2^). The red dashed line indicates the Fermi energy *E*_F_. In the DOS plot, pristine graphene is indicated with a continuous line, and the S-vacancy appears as a peak close to the Dirac point. (b) Field-effect-induced charge distribution as a function of gate voltage *V*_G_, evaluated as the difference between the gated (*V*_G_ ≠ 0) and ungated case (*V*_G_ = 0). The solid (dashed) red line indicates the excess holes on the graphene (MoS_2_) monolayer, while the solid (dashed) blue line indicates the excess electrons. (c, d) Side view of the gated graphene–MoS_2_ interface. In the two panels, the charge isosurface for *V*_G_ < 0 (left) and *V*_G_ > 0 (right) is evaluated as the difference between the charge densities for the gated and ungated limit. The location of the S-vacancy in the supercell is marked by the green ball.

**Figure 7:**
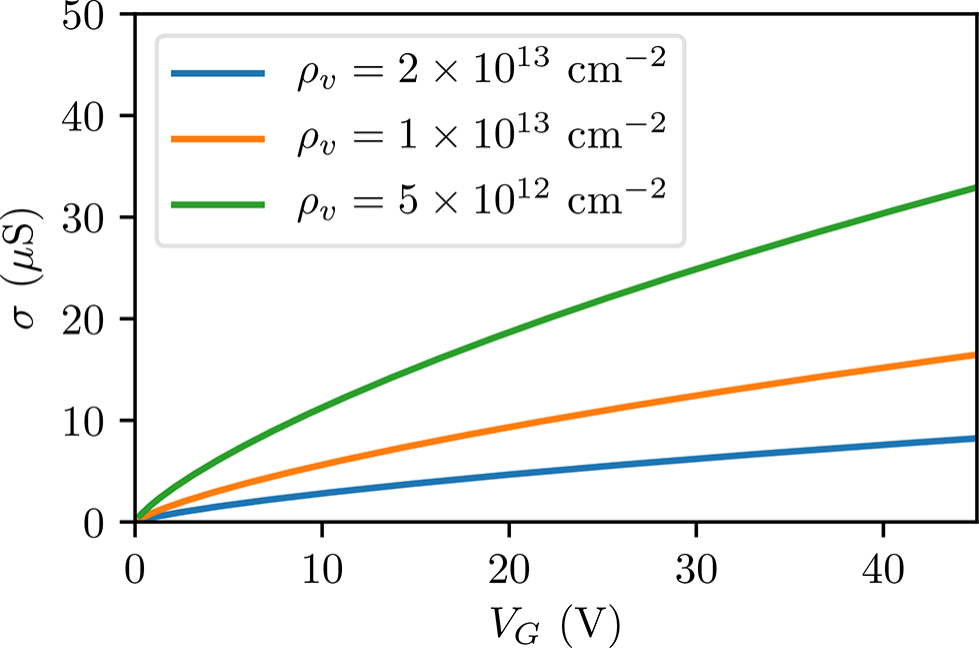
Quantitative estimate of the conductivity of graphene. Midgap states associated with sulfur vacancies can suppress mobility in graphene by increasing electron scattering. Conductivity suppression was calculated for three different densities of sulfur vacancies ρ_v_, using the carrier densities reported in Figure 6.

Using DFT,^55^ we made an *ab initio* analysis of the electronic states of graphene–MoS_2_^56^ in the presence of sulfur vacancies: These have an energy that falls in the gap^57^ of MoS_2_ and are located at a distance of few Angstroms from graphene, so their effect is hard to evaluate without a first-principles approach (see the “Methods” section and the Supporting Information for further details). Numerical calculations were performed using a density of S-vacancies of ρ_v_ ≈ 1.8 × 10^13^ cm^–2^. In Figure 6a, we report the electronic band structure and projected density of states (DOS) of the graphene–MoS_2_ heterostructure for *V*_G_ = 0: As visible in the plot, the Fermi energy of the system lays in the proximity of the MoS_2_ midgap states generated by the S-vacancies. This suggests that such states may influence the mobile carrier density induced in the graphene layer by the gate when *V*_G_ ≠ 0. This is confirmed by calculations performed at different values of *V*_G_. As shown in Figure 6b, even if MoS_2_ is placed on top of graphene, it affects the carrier density induced by the gate in the graphene layer. In particular, the n-side of the field-effect response is reduced by ∼50%; this, combined with the likely increased scattering^48,58,59^ caused by the large DOS close to the Fermi energy, clearly reproduces the behavior reported in Figure 5. The sulfur vacancies work as charge traps only for positive gate voltages and thus only for electron carriers. The sulfur vacancies generate a midgap state above the neutrality point, and scattering due to midgap states reduces the conductivity as described by the formula
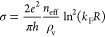
2where *n*_eff_ is the effective density of the charge carrier in the graphene sheet (∼50% of the induced doping charge), *k*_F_ is the Fermi momentum due to the effective density of the charge carrier *n*_eff_, ρ_v_ is the density of S-vacancies, and *R* = 3 Å is the average radius of the vacancy.^48^ In Figure 7, the quantitative estimation of the conductivity is shown. We used a range of ρ_v_ similar to the one used in the *ab initio* calculations. We plot the data only for positive gate voltages because the midgap states affect the transport properties only in that sector. In Figure 6c,d, the atomistic structure and the model of the typical setup for a field-effect measurement are shown. The charge density isosurfaces, together with the planar averaged carrier charge density, give a pictorial view of the different behavior with negative and positive backgate voltages. We point out that our results do not contradict, but rather complement, the conclusion drawn in recent literature:^22^ Calculations also show that no charge transfer is obtained in the case of defect-free MoS_2_ since in that limit the TMD cannot support any electron state in the relevant energy range (see the Supporting Information). Nonetheless, it is interesting to highlight that in the presence of vacancies a TMD overlayer can indeed have an impact on the backgate response of these vdW heterostructures and of devices based on them, extending the range of nontrivial consequences of the weak screening properties of graphene.

## Conclusions

We have demonstrated a graphene–MoS_2_ architecture integrating multiple graphene-contacted MoS_2_ FETs and MoS_2_-covered graphene FETs and used it to correlate the field-effect characteristics of a MoS_2_ monolayer with the conducting properties of graphene used to contact it. Such a study cannot be performed in a conventional FET structure since the individual resistive contributes cannot be discriminated in any obvious and direct way. Our results show that MoS_2_ can affect the field-effect conduction of a back-gated graphene monolayer, even when placed on top of the vdW stack, and the suppression of conduction in the graphene stripes is observed over a gate voltage range which is consistent with the conduction threshold of the MoS_2_ channel. This behavior is explained in terms of a filling of sulfur vacancies in the MoS_2_, as supported by *ab initio* calculations. The suppression of the electron transport can be exploited for the development of engineered optoelectronic devices based on van der Waals heterostructures,^10^ taking advantage of the low contact resistance of graphene in our multi-FET heterostructure.

## Methods

### Nanofabrication

The multi-FET fabrication starts from a square array of ∼150 μm wide single-crystal monolayer graphene flakes, with a spacing of 200 μm. Arrays are grown on Cu foil via CVD^30^ and then transferred on a p++ Si substrate covered by 300 nm thermal SiO_2_ using a delamination procedure and a semidry method based on a PMMA vector.^26^ Before the MoS_2_ transfer, the samples were cleaned from PMMA using an overnight immersion in acetone, followed by 2 min rinse in isopropanol, 3 min in AR 600–71 remover, and finally in deionized water. The next fabrication step was the patterning of graphene into a set of 5 μm wide and 5 μm spaced stripes. To this aim, we spun PMMA AR-P679.04 and baked the samples at 120 °C for 5 min. The stripe patterns were defined via electron-beam litography (EBL) using a SEM Zeiss Ultraplus with a Raith lithographic module, an energy of 20 keV and a dose of 300 μC/cm^2^. The samples were then developed in AR 600–56 for 2 min and a half. Then, graphene was etched by means of reactive ion etching (RIE) using Ar and O_2_ (5:80 sccm). Finally, the samples were again cleaned from PMMA with an overnight immersion in acetone and isopropanol rinsing. Single-crystal MoS_2_ monolayer flakes with an average size of 50 μm were grown via CVD following refs (60) and (61). Single-crystal monolayer MoS_2_ flakes were then transferred on the graphene stripes, using a semidry method.^60,61^ The transfer process employed for MoS_2_ is very similar to the one for graphene except for the delamination step, which was obtained by immersing the sample in a 1 M solution of NaOH rather than by an electrochemical method.^60^ Given the chosen spacing between the stripes, the process typically yields various devices with 4–5 contacts and, since the flakes are triangular, with an uneven coverage of the graphene stripes. A final post-transfer patterning was performed to remove excess material, using a laser writer Micro Writer ML3 and a S1818 photoresist mask with a 300 nm PMMA interlayer to protect the 2D materials form contamination by the photoresist. We then cleaned the samples with warm acetone (20 min) and chloroform for 1.5 h. To complete the devices, we defined a set of Cr/Au (10/50 nm) metallic electrodes, via EBL, evaporation, and lift-off. Using this method, 10 devices were fabricated in two batches and three multi-FETs were measured.

### Experimental Section

The properties of graphene and MoS_2_ were monitored by Raman and photoluminescence spectroscopy, using a Renishaw InVia spectrometer equipped with a 532 nm laser and a 100× objective lens (N.A. 0.85). Laser power was ∼1mW and the typical acquisition time was 4 s.^62^ Transport measurements were performed in a vacuum chamber using source-measure units K4200 and K2614B and a Femto DDPCA-300 current preamplifier.

### Numerics

We carried out DFT calculations by using QUANTUM ESPRESSO (QE),^63−67^ which uses a plane wave basis set. The pseudopotentials were taken from the standard solid-state pseudopotential (SSSP) accuracy library^68−72^ with increased cutoffs of 50 and 400 Ry for the wave functions and the density. The exchange-correlation potential was treated in the GGA, as parametrized by the Perdew–Burke–Ernzerhof (PBE) formula,^73^ with vdW-D2 correction as proposed by Grimme.^74^ For the BZ integrations, we employed a Marzari–Vanderbilt smearing^75^ of 10^–3^ Ry with a Monkhorst–Pack (MP)^76^***k***-point grid with 18 × 18 × 1 (24 × 24 × 1) points for self-consistent calculations of the charge density (density of states). The heterostructure of monolayer MoS_2_ on top of monolayer graphene is shown in Figure 6c,d, where an 8 × 8 MoS_2_ supercell is placed on a 10 × 10 supercell of graphene. The considered heterostructure model contains 391 (392) atoms in the unit cell for the simulation with (without) S-vacancy, corresponding to a density of S-vacancies of ρ_v_ ≈ 1.8 × 10^13^ cm^–2^. We keep the lattice constant of graphene unchanged at *a*_0_ = 2.46 Å^55,77^ and compressed the lattice constant of MoS_2_ by roughly ∼2.4%: from 3.15 Å^78^ to 3.075 Å. We considered a supercell with about 18 Å of vacuum along the *c*-direction between periodic images. We optimize the geometrical structures by relaxing only the atomic positions until the components of all the forces on the ions are less than 10^–3^ Ry/Bohr, while we keep fixed the lattice parameters.

In Figure 6c,d, a model of the typical setup for a field-effect measurement is shown. The graphene–MoS_2_ is placed in front of a metal gate. The layers are then charged with the same amount of opposite charge, leading to a finite electric field in the region between the heterostructure and the gate. In order to avoid spurious and artificial electric field between the different slabs of the repeated unit cell, an electric field generated by a dipole plate of opposite charge has been included next to the gate. Furthermore, to avoid the direct interaction between the charge-density of the system and the gate, a potential barrier has been included.^66,67^ In order to mimic the experimental values, in Figure 6b, we rescaled the values of *V*_G_ considering that in the real experiment there is a 300 nm thick layer of SiO_2_ between the metal gate and the graphene–MoS_2_ interface. We use the VESTA^79^ code to visualize the geometrical structure and the isosurfaces and to produce the plots in Figure 6. To obtain information on the charge transfer between the two moieties (graphene–MoS_2_), we performed a topological analysis of the electron density by means of the Bader procedure^80−83^ as implemented in CRITIC2.^84,85^

## Supporting Information Available

Additional Raman data, additional photoluminescence data, atomic force microscopy measurement, and additional DFT results (PDF)
