# Supplementary material for: Unexpected Electron Transport Suppression in a Heterostructured Graphene–MoS2 Multiple Field-Effect Transistor Architecture
Source: ACS Nano. 2021 Dec 23;16(1):1291–300. doi: 10.1021/acsnano.1c09131 (PMC8793137; doi:10.1021/acsnano.1c09131)
Supplement: Supplementary file 1 — nn1c09131_si_001.pdf [file nn1c09131_si_001.pdf]

Supporting Information: Unexpected Electron Transport Suppression in a Heterostructured Graphene-MoS_2 Multiple Field-Effect Transistor Architecture

Gaia Ciampalini,[*,†,‡,¶] Filippo Fabbri,[¶] Guido Menichetti,[‡,†] Luca Buoni,[†] Simona Pace,[‡,§] Vaidotas Mišeikis,[‡,§] Alessandro Pitanti,[¶] Dario Pisignano,[†,¶] Camilla Coletti,[‡,§] Alessandro Tredicucci,[†,¶] and Stefano Roddaro[†,¶]

†*Dipartimento di Fisica "E. Fermi," Università di Pisa, Largo B. Pontecorvo 3, I-56127 Pisa, Italy*

‡*Graphene Labs, Istituto Italiano di Tecnologia, Via Morego 30, I-16163 Genova, Italy*

¶*NEST, CNR—Istituto Nanoscienze and Scuola Normale Superiore, piazza San Silvestro 12, I-56127 Pisa, Italy*

§*Center for Nanotechnology Innovation @NEST, Istituto Italiano di Tecnologia, Piazza San Silvestro 12, I-56127 Pisa, Italy*

E-mail: gaia.ciampalini@phd.unipi.it

Contents

1 Additional Raman data **S-3**

1.1 Graphene S-3

1.2 MoS_2 . . . . S-6

2 Additional photoluminescence data **S-8**

3 Atomic Force Microscopy **S-9**

4 IV graphene stripes **S-10**

5 Effect of the electron transport suppression **S-11**

6 Additional DFT results **S-12**

7 Charge isosurfaces **S-16**

References **S-16**

1 Additional Raman data

1.1 Graphene

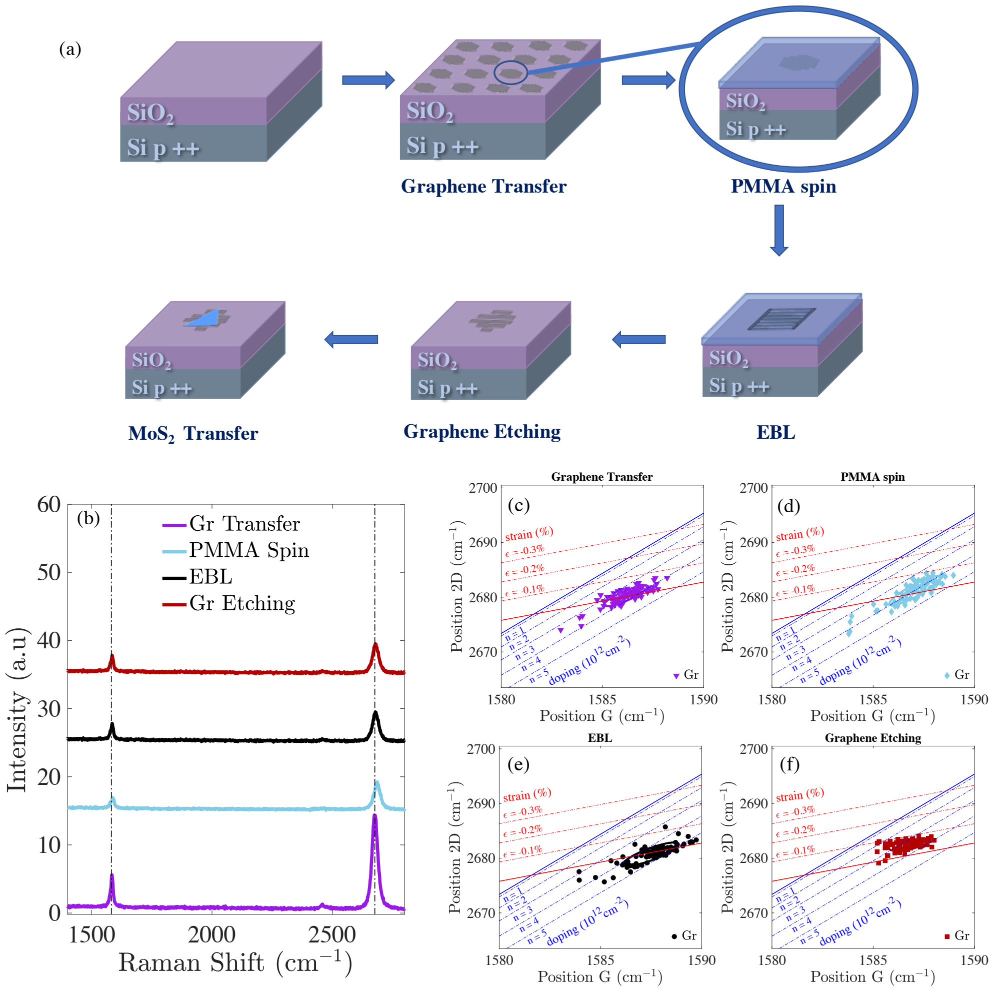

Figure S1: **Fabrication procedure and graphene characterization**. (a) Schematic of the fabrication procedure. (b) Graphene Raman spectra at different steps of fabrication. (c-f) Correlation plots of $2D$ position as a function of G position for each step of fabrication.

Raman spectroscopy allows to monitor the evolution of graphene properties throughout the fabrication process described in the sketches in Fig. S1 a. Fig. S1 b shows graphene Raman spectra at different steps of fabrication. In these spectra D peak is not measurable. Fig. S1c-f report the correlation plot of the position of $2D$ as a function of the position of G. From these plots, it is possible to estimate the strain and doping of the material. Linear fits give a slope $\Delta\text{Pos}(2D)/\Delta\text{Pos}(G) \approx 1.51, 1.93, 1.31, 0.61$ for graphene transfer, PMMA

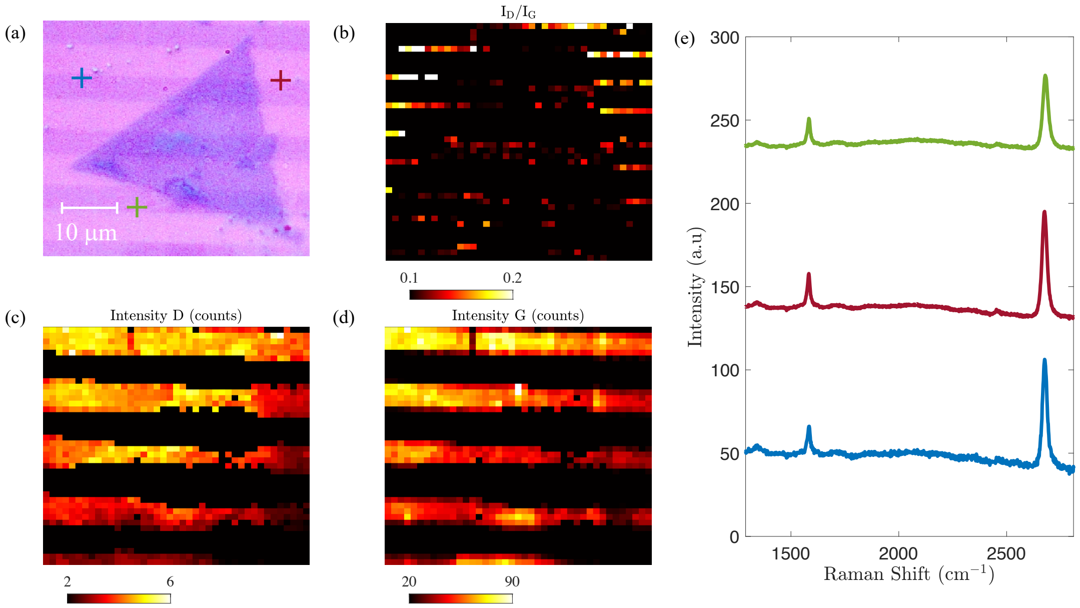

Figure S2: **Graphene Raman map** . (a) Optical image of the Graphene/MoS_2 heterostructure. (b) Ratio between the intensity of the D peak and the intensity of the G peak. (c) Intensity of the D peak. (d) Intensity of the G peak. (e) Selected spectra of the graphene stripes edges. Crosses in panel a identify spots where the spectra were taken. Color code in panel a is the same adopted in panel e.

spin, EBL and graphene etching respectively, indicating the presence of a non-zero strain and doping, depending on the specific step in the fabrication process. After the MoS_2 transfer (Fig 2 b of the main text) linear fits give a slope $\Delta\text{Pos}(2D)/\Delta\text{Pos}(G) \approx 1.53$ and 1.98 for the graphene under and outside of the MoS_2 flake respectively. Fig. S2 reports Raman maps of the I_D/I_G ratio (panel b), the D intensity (panel c), the G intensity (panel d) of the graphene stripes. Selected spectra of the stripes edges are reported in panel e, reporting the presence of D peak. Crosses in panel a identify spots where the spectra were taken. The intensity of the D peak is always faint and barely measurable above noise (less than 6 counts) even on the edge of the graphene stripes, as shown in panel e. Therefore, the higher ratio I_D/I_G at the edge of the mapped graphene stripes in panel b, is related, as expected, to the high concentration of defects at the graphene stripes edges. In Fig. S3, we reported all the maps for the G and $2D$ peaks parameters. Clearly, data show that the presence of the MoS_2 has a strong influence on the $2D$ peak, while the G peak is only slightly affected.

Such a behavior is confirmed by considering the histograms of Fig. S4, and in particular the panel (c) where we report the ratio between the intensity of the $2D$ peak and the intensity of the G peak: here two different groups of datasets can be clearly identified. One with a higher ratio, corresponding to the graphene outside of the MoS_2 flake, and another one with a lower value corresponding to the graphene under the flake.

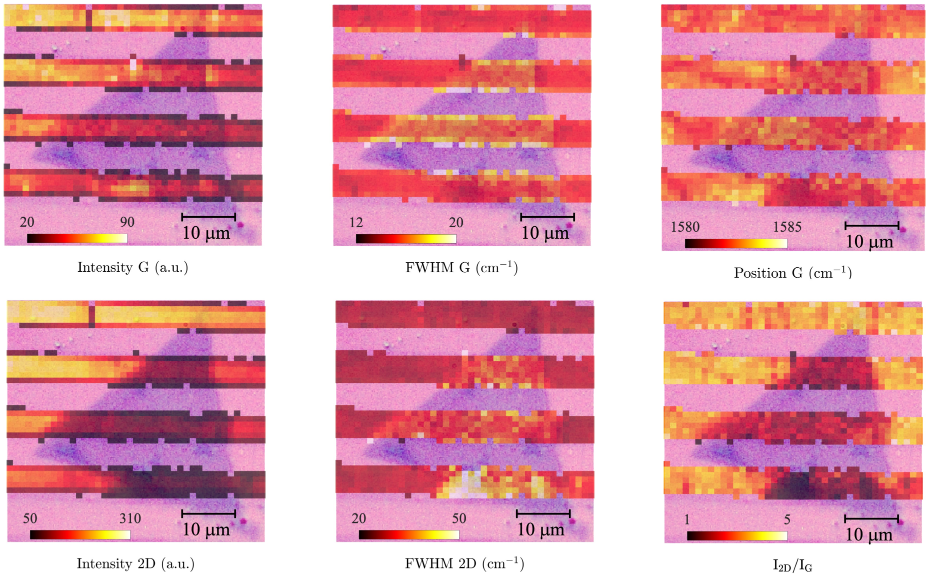

Figure S3: **Graphene Raman maps**. (a) Intensity of the G peak. (b) FWHM of the G peak. (c) Position of the G peak. (d) Intensity of the $2D$ peak. (e) FWHM of the $2D$ peak. (f) Ratio between the intensity of the $2D$ peak and the intensity of the G peak.

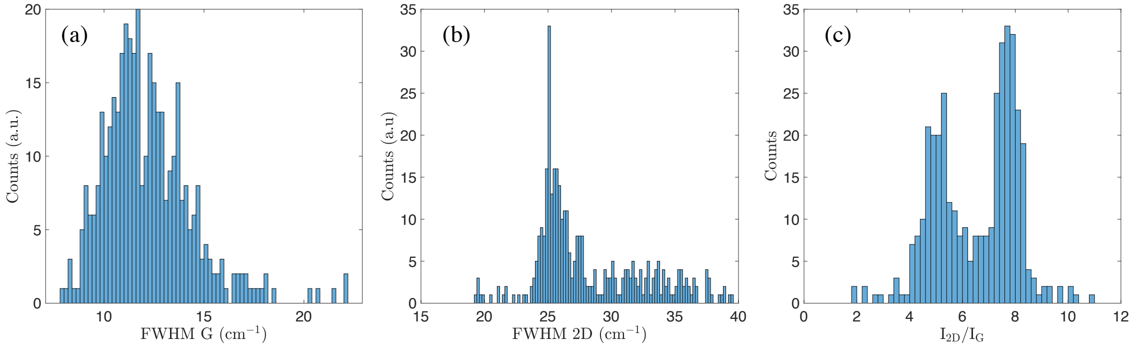

Figure S4: **Graphene Raman Histograms**. (a) FWHM of the G peak. (b) FWHM of the $2D$ peak. (c) Ratio between the intensity of the $2D$ peak and the intensity of the G peak.

1.2 MoS_2

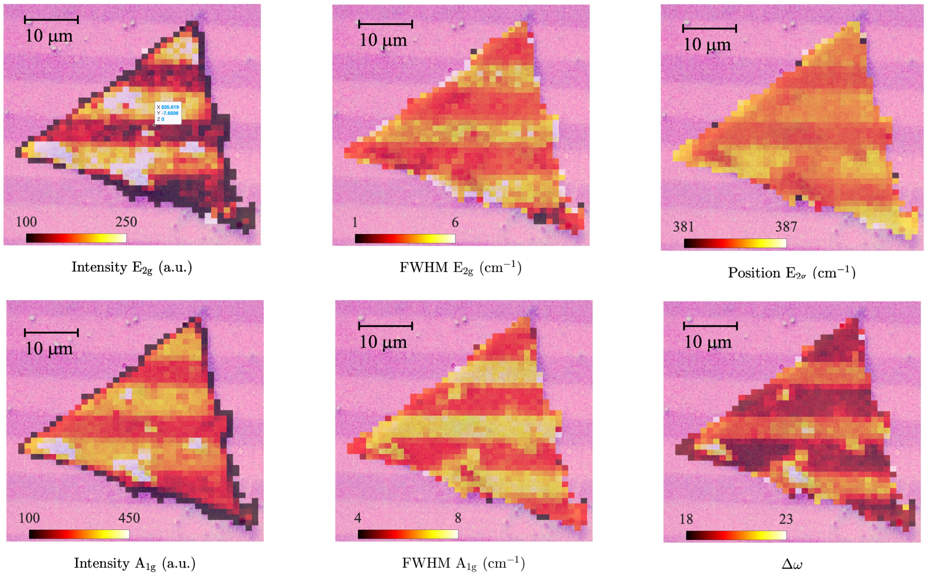

Figure S5: **MoS_2 Raman maps** . (a) Intensity of the E_{2g} peak. (b) FWHM of the E_{2g} peak. (c) Position of the E_{2g} peak. (d) Intensity of the A_{1g} peak. (e) FWHM of the A_{1g} peak. (f) Difference between the A_{1g} and E_{2g} positions.

MoS_2 Raman maps reported in Fig. S5a-e reveal that also as MoS_2 is affected by graphene. Fig. S5f shows the difference between the A_{1g} and E_{2g} peak positions. The $\Delta\omega$ map allows to precisely localize the bilayer islands. Representative Raman spectra from each region are reported in Fig. S6.

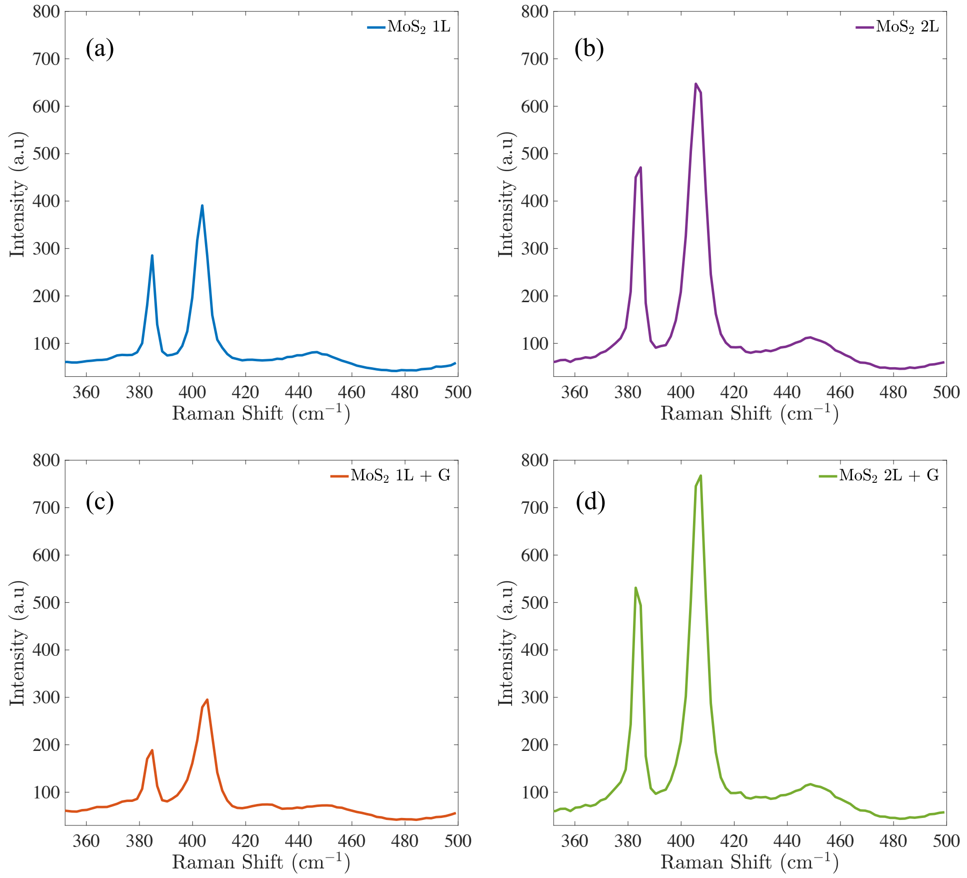

Figure S6: **MoS_2 Raman spectra from different regions** . (a) Monolayer MoS_2 spectrum. (b) Bilayer MoS_2 spectrum. (c) Monolayer MoS_2-graphene heterostructure spectrum. (d) Bilayer MoS_2-graphene heterostructure spectrum.

2 Additional photoluminescence data

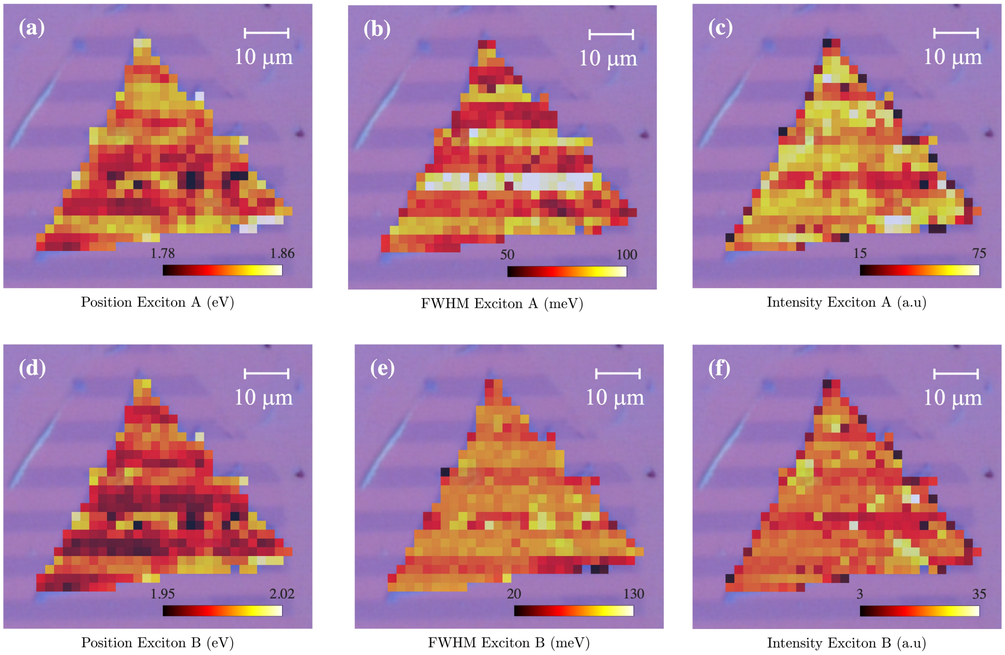

Figure S7: **MoS_2 PL maps** . (a) Position of the exciton A. (b) FWHM of the exciton A. (c) Intensity of the exciton A. (d) Position of the exciton B. (e) FWHM of the exciton B. (f) Intensity of the exciton B.

The effect of graphene on MoS_2 properties can be also seen in the MoS_2 PL spectra, as a suppression of the excition luminescence signal. The effect is stronger on the A exciton (see PL maps shown in Fig. 2e-f in the main text), but – though less severe – an effect is present also in the case of the B exciton. These measurements have been carried out on a different flake with respect to the one reported in the main manuscript.

3 Atomic Force Microscopy

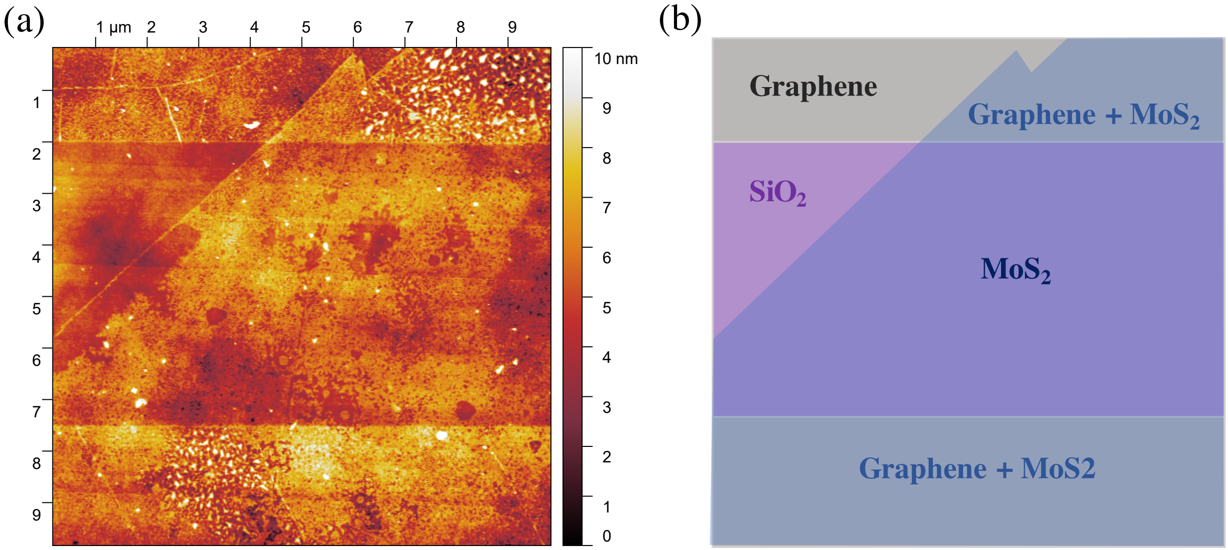

Figure S8: **AFM measurement** . (a) AFM topography. (b) Schematic of the investigated area.

Table S1: AFM parameters

	**Roughness (Rq) [nm]**	**Skewness**	**Kurtosis**
Graphene	1.00	1.61	12.97
Graphene + MoS_2	2.06	0.97	1.86
MoS_2	0.82	0.25	2.04
SiO_2	0.67	0.69	1.04

Fig. S8 shows an AFM topographic measurement of a region of the device including bare SiO_2, MoS_2, graphene as well as graphene-MoS_2 stacks, see sketch in panel (b). The topography reveals few wrinkles on the graphene stripes due to the transfer process and few holes in the MoS_2 flake. In addition some bubbles can be identified in the graphene-MoS_2 areas, probably due to a residual trapping of gas between the two monolayers.[S1] Table S1 reports the relevant parameters of the topography.

4 IV graphene stripes

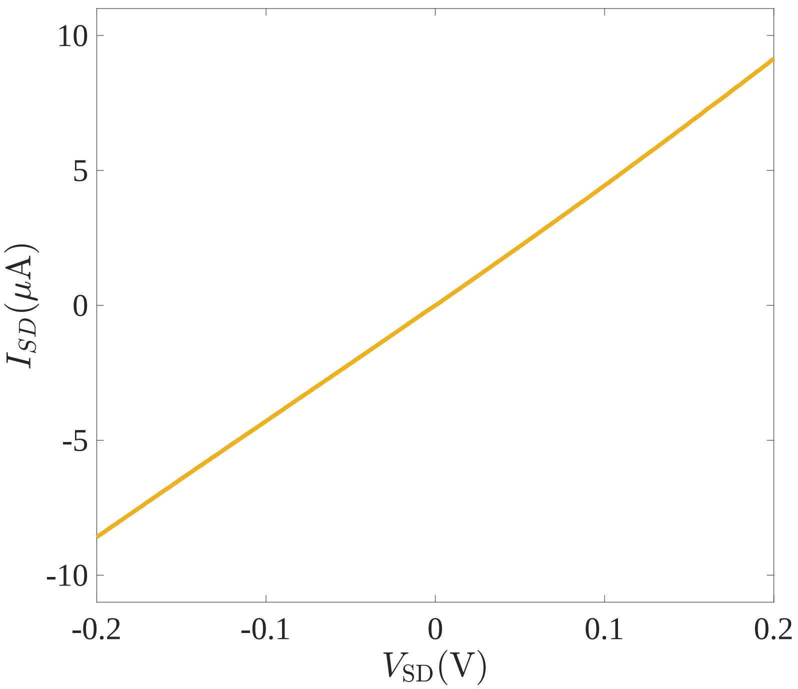

Figure S9: **IV graphene stripe** . A representative measurement to show the linearity of graphene IV. This measurement was carried out iat zero gate voltage, on the 69% MoS_2 covered stripe.

The IVs of all our graphene stripes are found to be highly linear even when graphene is covered by MoS_2, demonstrating that our graphene stripes operate in the linear response regime. Fig. S9 shows a representative measurement obtained at zero gate voltage, for the stripe with 69% MoS_2 coverage.

5 Effect of the electron transport suppression

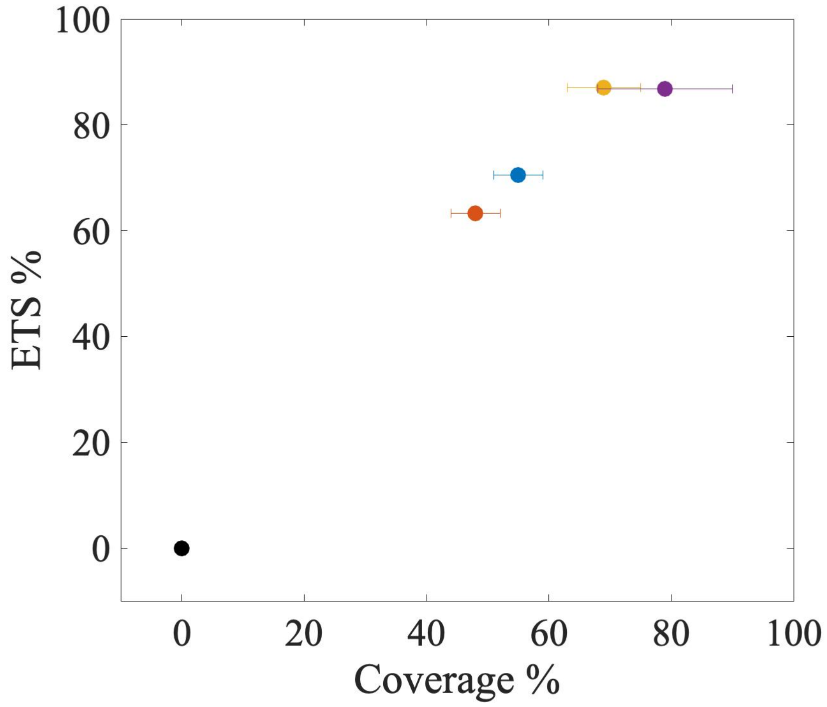

Figure S10: **Electron transport suppression (ETS).** Percentage of the electron conductance suppression as a function of the MoS_2 coverage values.

In Fig. S10, the Electron Transport Suppression (ETS) was calculated by comparing the conductivity of MoS2-covered stripes with the conductivity of the MoS2-free stripes, for each of the studied coverage percentages, using the highest available positive gate voltage (60V). The uncertainty in the coverage percentage is calculated based on the precision of the optical imaging. Fig. S10 shows an approximately linear dependence of the electron conductance suppression as a function of the MoS_2 coverage values.

6 Additional DFT results

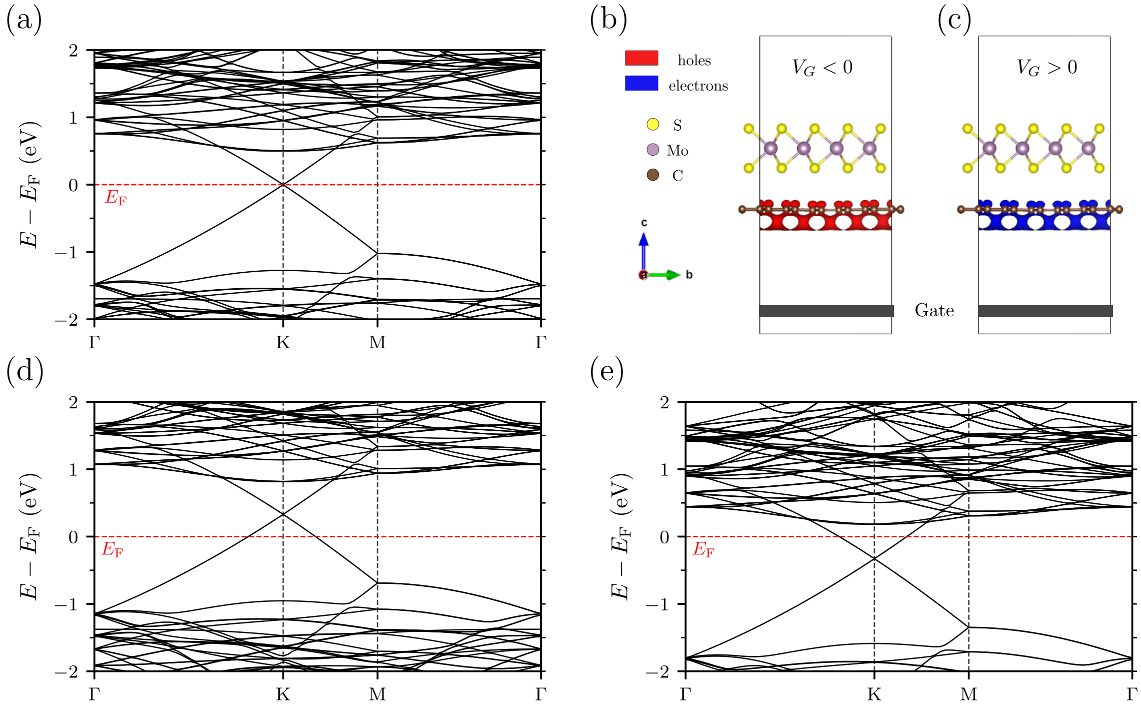

Figure S11: **Field effect response in the absence of S-vacancies**.In the absence of S-vacancy we used a supercell composed by a 4×4 MoS_2 supercell placed on a 5×5 supercell of graphene. (a) Electronic band structure of a Graphene-MoS_2 interface in the absence of S-vacancies, in the undoped limit. The red dashed line indicates the Fermi energy E_F. (b-c) Side views of the Graphene-MoS_2 interface placed in front of a metal gate. The Isosurfaces of the charge distribution induced by field effect for $V_G < 0$ (panel b) and $V_G > 0$ (panel c) are evaluated as difference between the full charge density of the doped system and the undoped system. The configurations correspond to $V_G = \pm 45$ V where the doping charge is $n \approx \pm 1.2 \times 10^{13}\ cm^{-2}$, respectively. (d-e) Electronic band structure of Graphene-MoS_2 interface without S-vacancies, for the configuration considered also for the panel (b) and (c) ,respectively. The red dashed line indicates the Fermi energy E_F. The isosurface in figure corresponds to a value of charge density of $\approx 6.7 \times 10^{-4} e$ Å^{-3}

In order to support our conclusions and compare them with existing literature, we carried out DFT calculations also in the case of defect-free MoS_2. In the absence of S-vacancies, we demonstrate that no asymmetry is obtained between electron and hole doping by field effect. In fact, in the neutral case the Dirac cone lies deep in the gap of MoS_2 (as shown in Fig. S11a) and the charge induced by field effect in the proximity of E_F is not influenced by the MoS_2 layer. To confirm such a conclusion, we studied the system under multiple gate potentials

($V_G > 0$ and $V_G < 0$). In particular, in Fig. S11b-c we show results for the $V_G = \mp 45\,\mathrm{V}$ cases: the charge induced by field effect is $n \approx \pm 1.2 \times 10^{13}\,\mathrm{cm}^{-2}$, respectively, and is spatially localized only on the graphene monolayer. Also the electronic band structures for such cases, as shown in Fig. S11d-e, confirm this picture. In fact, the field-induced charge is simply associated with the filling (or depletion) of the Dirac cone in the graphene monolayer.

Conversely, in the presence of S-vacancies, the field-effect response of the electronic band structures, as shown in Fig. S13a-b, is asymmetric on the hole (S13a, $V_G < 0$) or electron (S13b, $V_G > 0$) side of the field effect response. Comparing Fig. S13b with Fig. S11e, we can see that for $V_G > 0$ the Dirac cone is less populated and the Fermi energy partially overlaps with the states associated with the S-vacancy. This happens because only a portion of the carrier density lies on the graphene layer, while the missing charge is accumulated around the S-vacancies, as clearly depicted in Fig. S13d. Such charge is effectively "trapped" and does not contribute to the electronic transport because the S-vacancy state is very narrow/dispersionless, indicating it is very localized. Comparing Fig. S11d with Fig. S13a, and Fig. S11b with Fig. S13c (*i.e.* the cases of hole doping, without or with defects, respectively) we can see that graphene behaves in an equivalent way regardless the presence/absence of S-vacancies: in both cases they do not affect the field effect on the hole side of the Dirac cone. We performed our calculations with a density of S-vacancies of $\rho_v \approx 1.8 \times 10^{13}\,\mathrm{cm}^{-2}$, which corresponds to one S-vacancy in the simulation cell, *i.e.* we removed one sulfur atom in a 8×8 MoS_2 supercell. In the plots, the vacancy is located in the sulfur plane closest to the graphene layer, but the position (closer or further away from graphene) of the vacancy is not found to affect the conclusions of our calculations. We also verified how this result depends on the density of S-vacancies. In particular we repeated the analysis with $\rho_v \sim 2.5 \times 10^{13}$ cm^{-2}, $\rho_v \sim 3.8 \times 10^{13}$ cm^{-2} and $\rho_v \sim 7.5 \times 10^{13}$ cm^{-2}. The field-effect induced charge distributions do not change with increasing density of S-vacancies, in fact in Fig. S12 we can see that, despite the increasing density of S-vacancies, almost the same fraction of electrons goes on the MoS_2 monolayer.

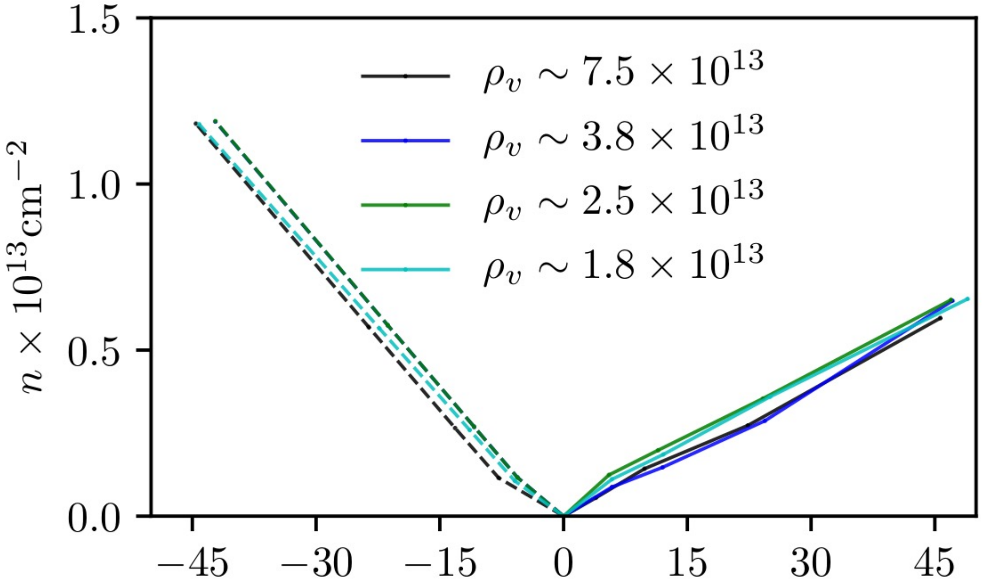

Figure S12: **Field-effect induced charge distribution with an increasing density of S-vacancies**. Field-effect induced charge distribution as a function of gate voltage V_G, evaluated as the difference between the gated ($V_G \neq 0$) and ungated case ($V_G = 0$); the topological analysis of the electron density was done by means of the Bader theory as discussed in the main text. The colored solid (dashed) lines indicate the excess electrons (holes) on the graphene monolayer, while the color density of S-vacancies. In order to mimic the experimental values, the values of V_G are rescaled considering that there is a 300 nm thick layer of SiO_2 between the metal gate and the Graphene-MoS_2 interface.

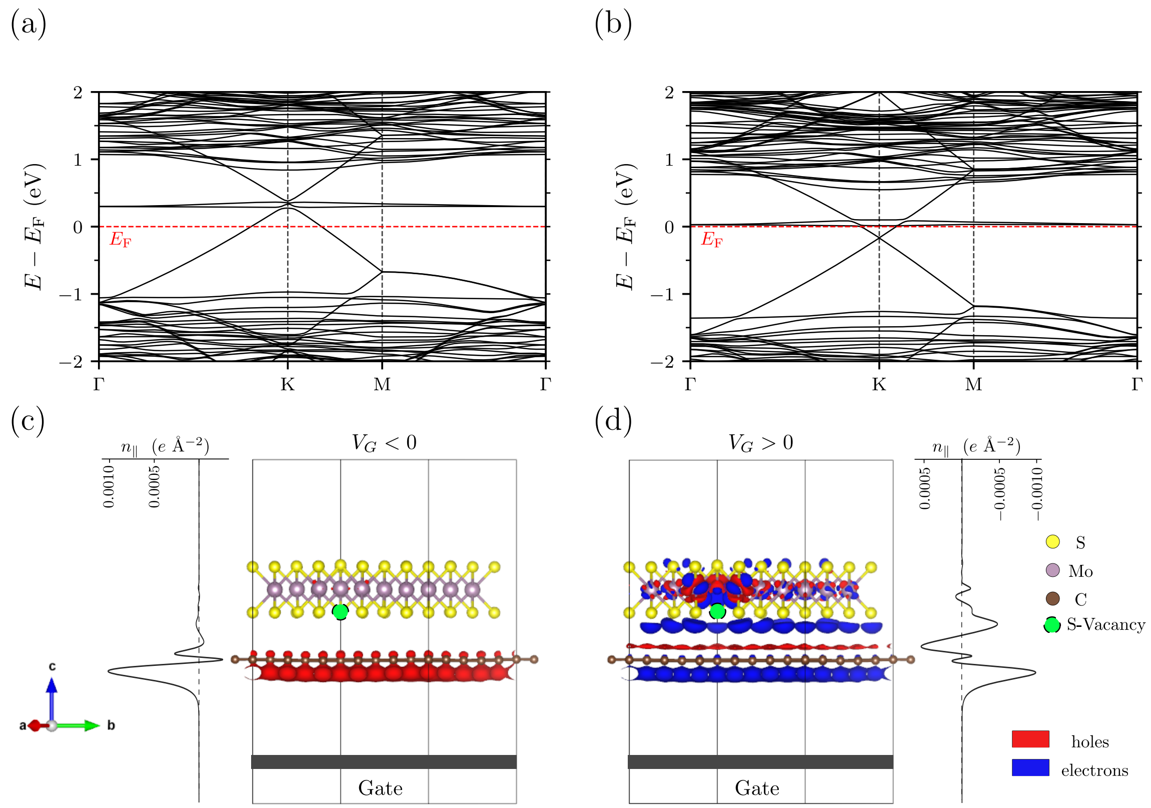

Figure S13: **Field effect on the electronic band structure and the charge distribution**. (a) Electronic band structure of the graphene-MoS_2 interface with S-vacancies. a field-induced charge $n \approx 1.2 \times 10^{13}\,\mathrm{cm}^{-2}$ is obtained for $V_G \approx -45\,\mathrm{V}$ (see Fig 3b). In this configuration, the S-vacancy state is far from the Fermi energy and it does not contribute to the transport nor it affects the gating. (b) The same situation for $V_G \approx +45\,\mathrm{V}$ leads to a total field-induced charge of $n \approx -1.2 \times 10^{13}\,\mathrm{cm}^{-2}$. In this case, the proximity of the Fermi energy to the S-vacancy state implies a significant part of the field-induced charge ends on mid-gap states created by the S-vacancy, in the MoS_2. The red dashed lines indicate the Fermi energy E_F. (c-d) Side view of the gated graphene-MoS_2 interface. In the two panels, the charge isosurface for $V_G < 0$ (left) and $V_G > 0$ (right) is evaluated as the difference between the charge densities for the gated and ungated limit; the location of the S-vacancy is marked by the green ball. The isosurface corresponds to a charge density of $\approx 6.7 \times 10^{-4} e\,\text{Å}^{-3}$. To compare these results with the ones in Fig. S11, the impact of sulfur vacancies was simulated by removing one S atom from a 4×4 MoS_2 supercell placed on a 5×5 supercell of graphene which corresponds to a density of S-vacancies of $\rho_v \sim 7.5 \times 10^{13}\,\mathrm{cm}^{-2}$. These results do not depend on the density of S-vacancies, in the range we have explored.

7 Charge isosurfaces

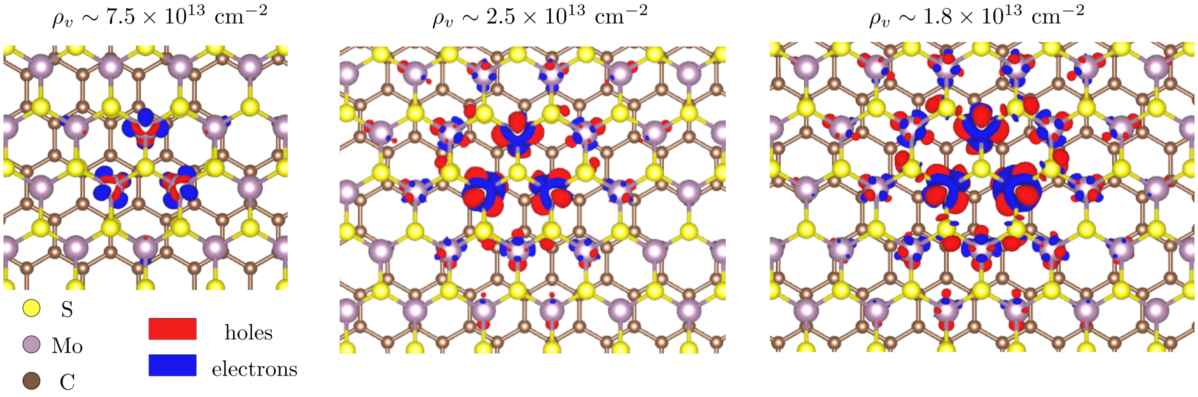

Figure S14: **Charge isosurfaces of the field-effect induced charge accumulation with a decreasing density of S-vacancies.**

Top view of the charge isosurfaces for and $V_G > 0$ are evaluated as the difference between the charge densities for the gated ($V_G \neq 0$) and ungated case ($V_G = 0$). The blue (red) isosurfaces are for negative (positive) accumulated charge. It is evident that the induced charge accumulates around the S-vacancy and the radius of the induced charge distribution increases with decreasing the density of S-vacancies, while the integral is almost the same (see the right side of Fig. S12). To properly compare the three cases, the charge isosurface level has been chosen the same for all the cases.

References

(S1) Khestanova, E.; Guinea, F.; Fumagalli, L.; Geim, A. K.; Grigorieva, I. V. Universal Shape and Pressure inside Bubbles Appearing in van der Waals Heterostructures. *Nat. Commun.* **2016**, *7*, 1–10.
